# Concomitant pulmonary artery and aortic embolectomy

**DOI:** 10.1186/1749-8090-8-S1-P13

**Published:** 2013-09-11

**Authors:** P Schraverus, JF Adam

**Affiliations:** 1 Cardio-Vascular Surgery, Grand Hopital De Charleroi, Charleroi, Belgium; 2 Intensive Care, Clinique Notre-Dame de Grâce, Gosselies, Belgium

## 

We report a case of a young patient presenting concomitant pulmonary artery embolism and an intra-aortic thrombus occluding the left subclavian artery due to a patent foramen ovale. This patient was treated under short circulatory arrest and unilateral cerebral perfusion allowing aortic and pulmonary embolectomy and closure of the small PFO. Follow up was uneventful so this case shows a successful treatment of a massive paradoxical aortic embolism and pulmonary embolism. Complete preoperative imaging is necessary to choose the safest cannulation option.

